# PIWIL1 interacting RNA piR-017724 inhibits proliferation, invasion, and migration, and inhibits the development of HCC by silencing PLIN3

**DOI:** 10.3389/fonc.2023.1203821

**Published:** 2023-07-11

**Authors:** Yi-Jing Wu, Jie Wang, Peng Zhang, Liu-Xia Yuan, Lin-Ling Ju, Hui-Xuan Wang, Lin Chen, Ya-Li Cao, Wei-Hua Cai, Yi Ni, Min Li

**Affiliations:** ^1^ Medical School of Nantong University, Nantong Third People’s Hospital, Affiliated Nantong Hospital 3 of Nantong University, Nantong, China; ^2^ Nantong Institute of Liver Disease, Department of Hepatobiliary Surgery, Nantong Third People’s Hospital, Affiliated Nantong Hospital 3 of Nantong University, Nantong, China; ^3^ Nantong Institute of Liver Disease, Nantong Third People’s Hospital, Affiliated Nantong Hospital 3 of Nantong University, Nantong, China; ^4^ Preventive Health Department, Nantong Third People’s Hospital, Affiliated Nantong Hospital 3 of Nantong University, Nantong, China; ^5^ Department of Hepatobiliary Surgery, Nantong Third People’s Hospital, Affiliated Nantong Hospital 3 of Nantong University, Nantong, China; ^6^ Thyroid and Breast Surgery, Nantong Third People’s Hospital, Affiliated Nantong Hospital 3 of Nantong University, Nantong, China; ^7^ Integrating Traditional Chinese Medicine with Hepatology, Nantong Third People’s Hospital, Affiliated Nantong Hospital 3 of Nantong University, Nantong, China

**Keywords:** hepatocellular carcinoma, piR-017724, proliferation, migration, invasion, PLIN3

## Abstract

**Background:**

Hepatocellular carcinoma (HCC) accounts for the majority of primary liver cancers. Worldwide, liver cancer is the fourth most common cause of cancer-related death. Recent studies have found that PIWI-interacting RNAs (piRNAs) participate in the occurrence and development of various tumors and are closely related to the growth, invasion, metastasis and prognosis of malignant tumors. Studies on the role and functional mechanism of piRNAs in HCC development and progression are limited.

**Methods:**

Quantitative reverse transcription-polymerase chain reaction (qRT-PCR) were used to detect the expression of piR-017724 in both HCC tissues and cells. Based on the clinical data of HCC patients, the clinical and prognostic value of piR-017724 was further analyzed. Then, targeted silencing and overexpressing of piR-017724 in HCC cells was further used to examine the biological functions of piR-017724. In addition, the downstream target protein of piR-017724 was predicted and validated through high-throughput sequencing and public databases.

**Results:**

The piR-017724 was significantly downregulated in HCC tissues and cells, and the downregulation of piR-017724 was associated with tumor stage and poor prognosis in HCC. The piR-017724 inhibitor promoted the proliferation, migration and invasion of HCC cells, while the piR-017724 mimic had the opposite effect. However, the piR-017724 did not affect apoptosis of HCC cells. High-throughput sequencing and qRT-PCR confirmed a reciprocal relationship between piR-017724 and PLIN3. Therefore, we speculate that piR-017724 may inhibit the development and progression of HCC by affecting the downstream protein PLIN3.

**Conclusions:**

Our study shows that piR-017724, which is lowly expressed in HCC, inhibits the proliferation, migration and invasion of HCC cells and may affect the development of hepatocellular liver cancer through PLIN3, which provides new insights into the clinical application of piR-017724 in the treatment of hepatocellular carcinoma.

## Introduction

Liver cancer is one of the most common malignancies worldwide and is also a leading cause of cancer-related death (1). The proportion of hepatocellular carcinoma (HCC) among primary liver cancer cases worldwide is 75%-85%, while this rate has increased sharply to 93.0% in China (2, 3). Risk factors for hepatocellular carcinoma include autoimmune hepatitis, alcoholism, diabetes, and chronic hepatitis B virus (HBV) infection (4–6). Although there are many immunotherapies and targeted therapies for HCC, HCC patients generally still have poor prognosis with a high probability of postoperative recurrence and metastasis (7–9). Because of the high rate of recurrence and metastasis, HCC patients are often diagnosed with a poor prognosis, especially in the terminal stages (10). HCC involves multiple changes in gene expression; therefore, understanding these changes is important for identifying new and significant genes in the pathogenesis of the disease. This diversity could help promote promising therapies for HCC. Therefore, to develop new therapeutic strategies to reduce the mortality of this malignancy, it is necessary to further elucidate the molecular pathogenesis of HCC.

PIWI-interacting RNAs (piRNAs) have become a new research hotspot in recent years. piRNA is a noncoding RNA whose length is approximately 26 to 31 nucleotides. It interacts with PIWI protein family members specifically expressed in germ cells to form the piRNA silencing complex (piRISC). piRNA/PIWI complexes affect transposon silencing, spermatogenesis, genome rearrangement, epigenetic regulation, protein regulation, and germline stem cell maintenance (11). Although little is known about the function of piRNAs in human cancer, emerging studies suggest that piRNAs may play an important role in cancer development and progression and may serve as diagnostic and prognostic biomarkers (12–15).

In the current work, we have studied the expression level of hsa_piR_017724 in HCC and its correlation with prognosis for the first time, and we have proposed the downstream PLIN3 network of hsa_piR_017724 to further explore its potential role in the pathogenesis of HCC.

## Materials and methods

### Patients and samples

Neoplastic liver tissues and matched adjacent nonneoplastic liver tissues were collected from 45 patients undergoing radical hepatocellular carcinoma surgery at the Nantong Third People’s Hospital. Informed consent was obtained from recruited patients, and the study protocol was approved by the Clinical Research Ethics Committees of Nantong Third People’s Hospital. All cases were reviewed by experienced pathologists who confirmed the diagnosis of HCC. Patient statistics are listed in **Table 1**.

**Table 1:** Clinical characteristics of the included patients.

Clinical characteristic	No. (%) or median (IQR)
Sex	
Male	35 (79.55%)
Female	9 (20.45%)
Age	
≥60	20 (45.45%)
<60	24 (54.55%)
Tumor size, cm	4.28 ± 2.20
Tumor stage	
I-II	12 (27.27%)
III-IV	32 (72.73%)
Vascular invasion	
Yes	13 (29.55%)
No	7 (15.91%)
Missing	24 (54.54%)
AFP, ng/mL	147.50 [3.68, 1348.72]
ALT, U/L	28.50 [19.00, 46.25]
AST, U/L	31.00 [26.75, 46.50]

### Cell culture

Human HCC cell lines (SMMC-7721, Li-7, PLC/PRF/5, SK-HEP-1, Huh-7, MHCC-97H, HCC-LM3) and the normal human hepatic cell line (L‐02) were purchased from Shanghai Cell Bank. The normal human hepatic cell line (L‐02) and human HCC cell lines (SMMC-7721, Li-7) were cultured in RPMI‐1640 (Gibco, Thermo Fisher Scientific, Waltham, MA). Human HCC cell lines (PLC/PRF/5, SK-HEP-1) were cultured in MEM (supplemented with NaHCO3 and sodium pyruvate). Human HCC cell lines (Huh-7, MHCC-97H, HCC-LM3) were cultured in Dulbecco’s modified Eagle’s medium (Gibco, Thermo Fisher Scientific). All the media contained 10% fetal bovine serum (FBS; Cell Sciences, Canton, MA). All cells were cultured at 37°C in a humidified incubator under 5% CO2 conditions.

### Quantitative reverse transcription-polymerase chain reaction

Total RNA from cell lines and clinical tissues was extracted with TRIzol reagent. To quantify piRNAs, real-time qPCR was performed using the Bulge-LoopTM qPCR kit (RiboBio, Guangzhou, China) according to the manufacturer’s protocol. U6 small nuclear RNA was used as an internal control in both cell lines. All experiments were performed in three biological replicates. The relative expression of RNA was calculated as the power value (2-△△Ct).

### Cell transfection

The hsa_piR_017724 mimic, the hsa_piR_017724 inhibitor and the corresponding negative control were synthesized by RiboBio (Guangzhou, China). SMMC-7721 and PLC/PRF/5 cells were seeded in 6-well plates and cultured overnight for transfection. After mixing the two solutions, the mixture was added to a 6-well plate according to the manufacturer’s instructions. Cells were harvested 48 hours after transfection, and the efficiency was verified by PCR.

### Cell viability assays

Cell viability was monitored using a Cell Counting Kit-8 (CCK-8, Dojindo, Japan). In brief, 48 h after transfection, HCC cells were seeded into 96-well plates at a density of 3×10^3 cells per well in 100 μl medium and cultured overnight. Cell viability was measured at 2.5 hours after the addition of CCK-8 reagent every 24 hours at OD450.

### Colony formation assays

Transfected HCC cells were trypsinized and resuspended to obtain a single cell suspension. Cells were counted and seeded into 6-well plates at a density of 2000 cells per well. The medium was changed every three days, and the cells were cultured for 2 weeks to obtain colonies. Colonies were washed with phosphate-buffered saline (PBS), fixed with 4% paraformaldehyde fixative, and stained with crystal violet (Beyotime). After washing and air drying, cell clones were photographed and counted.

### Cell apoptosis analysis by flow cytometry

For the cell apoptosis assay, an Annexin V/7-AAD Apoptosis Detection Kit (BD) was used to stain cells. Both experiments were assessed by flow cytometry using FlowJo software (BD).

### Transwell assay

Cell invasion and migration abilities were analyzed by 24-well transwell chambers (Corning, NY, USA) with 8.0 μm pores. At 48 hours after transfection, SMMC-7721 cells and PLC/PRF/5 cells were harvested by trypsin and then suspended in serum‐free medium. The cells were seeded onto the upper chamber at a density of 2×10^5, and the appropriate complete medium with 20% FBS was added to the bottom chamber. After culturing for 24 h in a 37°C, 5% CO2 incubator, cells that stayed in the upper chamber were removed. Cells that migrated through the filter were fixed with 4% paraformaldehyde, washed with PBS, and stained with crystal violet for 8 minutes. For invasion experiments, transwell membranes were precoated with Matrigel (BD Biosciences, San Jose, CA, USA). The number of cells that migrated or invaded was counted in three random fields by using an optical microscope (Olympus, Japan).

### Identification of downstream target proteins for piR-017724

The potential target proteins of piR-017724 were predicted by high-throughput sequencing technology combined with bioinformatics analysis, and the prediction results were verified by qRT-PCR.

### Statistical analysis

All experiments were performed at least three times. Statistical analysis of all experimental data was performed using SPSS software (version 25.0). GraphPad Prism (version 8.0) was used to determine statistical results. P<0.05 was considered statistically significant.

## Results

### The piR-017724 is significantly downregulated in HCC tissues and cell lines

**Figure 1:**
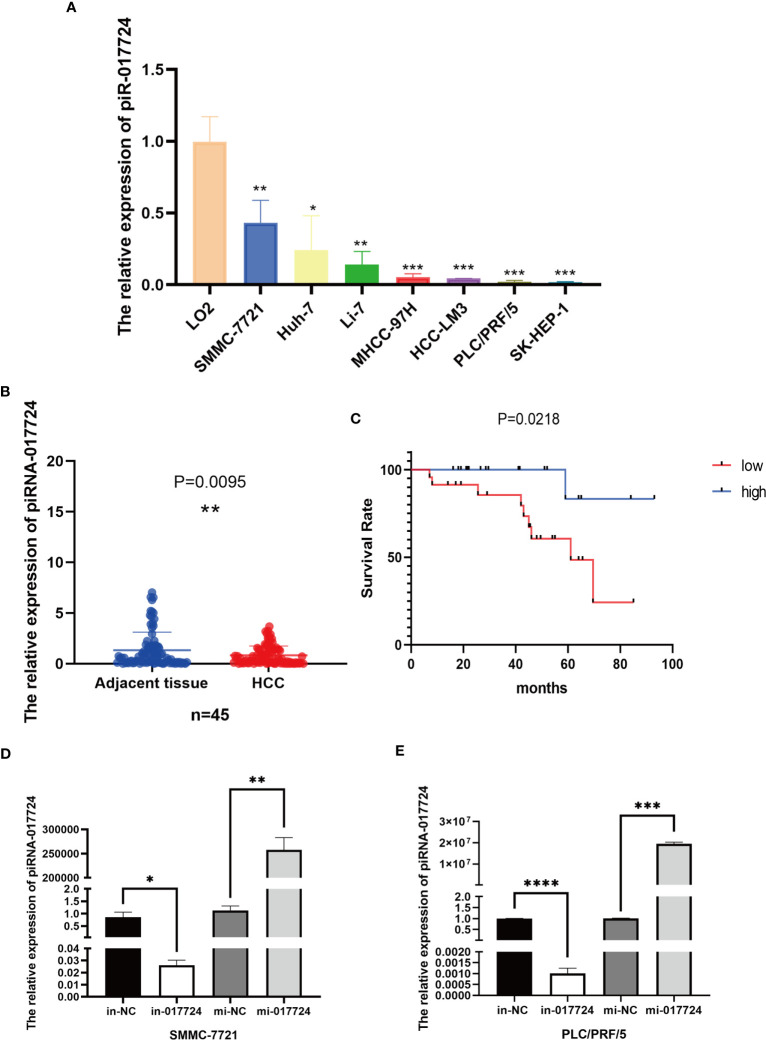
The relative expression of piR-017724. **(A)** Detection of piR-017724 expression using qRT-PCR in HCC cell lines and in normal human liver HL-7702 (L-02) cells, which served as the control. **(B)** Detection of piR-017724 expression using qRT-PCR in HCC (n=45) and adjacent tissues (n=45). **(C)** Kaplan–Meier plots of the overall survival of 45 patients with HCC stratifed by piR-017724 expression. **(D)** The transfection efficiency of SMMC-7721 cells was detected by qRT−PCR. **(E)** The transfection efficiency of PLC/PRF/5 cells was detected by qRT−PCR. *P < 0.05, **P < 0.01, ***P <0.001, ****P < 0.0001.

First, we verified the expression of piR-017724 in HCC tissues and cell lines by RT−PCR. Compared with normal hepatocytes, we found that piR-017724 levels were significantly downregulated in HCC cells (**Figure 1A**). Similarly, we found that piR-017724 was expressed at low levels in 45 pairs of liver cancer tissues (**Figure 1B**).

### The piR-017724 downregulation is associated with high malignancy and poor prognosis in HCC patients

Based on the expression data of piR-017724, we divided the cases into piR-017724-high and piR-017724-low groups. We further analysed the correlations between piR-017724 expression and diferent clinical features (**Table 2**). The results showed that the expression level of piR-017724 was significantly correlated with the malignant progression of HCC. The piR-017724 expression in patients with advanced HCC (stage I-II) was signifcantly higher than that in patients with early HCC (stage III-IV).

**Table 2:** Correlation between piR-017724 and clinical characteristics.

Clinical characteristic	piR-017724 expression		*P*-value
	Low (n=22)	High (n=22)	
Sex			1.000
Male	17 (77.27%)	18 (81.82%)	
Female	5 (22.73%)	4 (18.18%)	
Age			0.069
≥60	7 (31.82%)	13 (59.09%)	
<60	15 (68.18%)	9 (40.91%)	
Tumor size, cm	3.64 ± 2.02	4.92 ± 2.23	0.052
Tumor stage			0.042
I-II	3 (13.64%)	9 (40.91%)	
III-IV	19 (86.36%)	13 (59.09%)	
Vascular invasion			0.642
Yes	5 (22.73%)	8 (36.36%)	
No	4 (18.18%)	3 (13.64%)	
Missing	13 (59.09%)	11 (50.00%)	
AFP, ng/mL	147.40 [3.23, 1046.32]	190.32 [3.94, 2234.50]	0.778
ALT, U/L	33.00 [17.00, 52.50]	27.00 [20.00, 46.50]	0.900
AST, U/L	31.00 [26.00, 51.50]	31.00 [26.50, 44.00]	0.867

P-value ≤ 0.05 indicate statistical significance.

The survival analysis showed that the average survival time of 22 patients with low piR-017724 expression was 59.98 ± 5.94 months and that of 22 patients with high piR-017724 expression was 87.33 ± 5.17 months. The piR-017724 expression was associated with the survival of patients with HCC. The prognosis of patients with HCC presenting low piR-017724 expression was signifcantly poor (**Figure 1C**).

### The piR-017724 inhibits the proliferation of HCC cells

**Figure 2:**
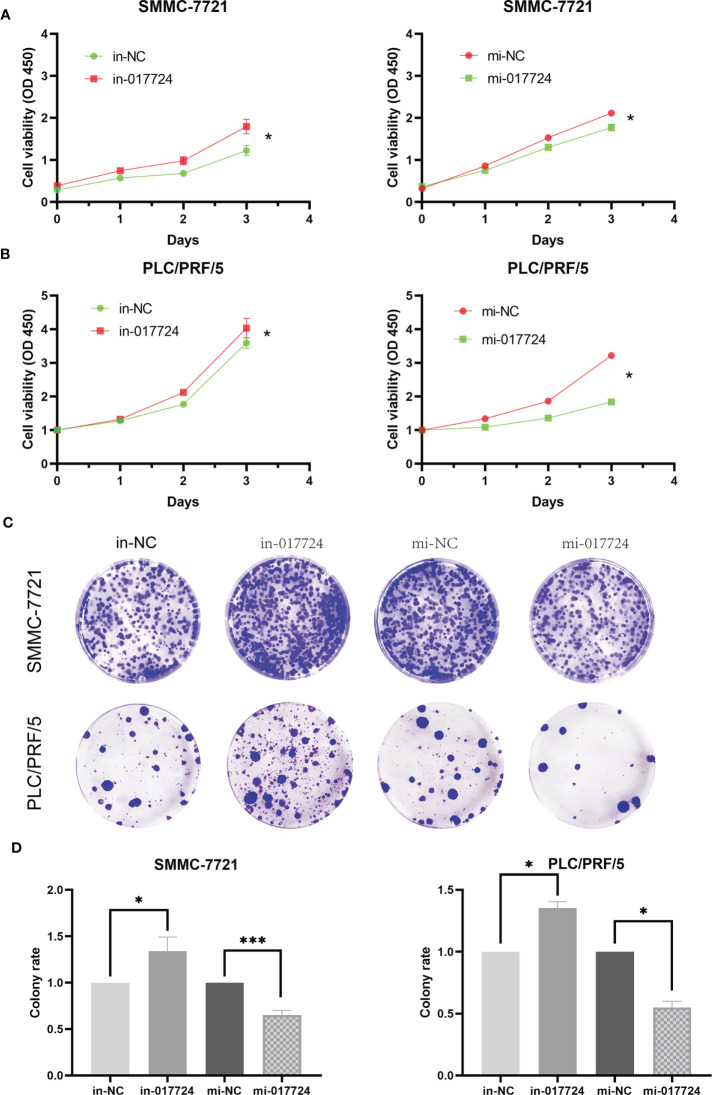
The piR-017724 inhibits the proliferation of HCC cells. **(A)** CCK-8 assay was applied to determine the proliferative ability of cells when piR-017724 knocked down and overexpressed in SMMC-7721 cells. **(B)** CCK-8 assay was applied to determine the proliferative ability of cells when hsa_piR_017724 knocked down and overexpressed in PLC/PRF/5 cells. **(C, D)** Colony formation assay was used to assess cell proliferation. *P < 0.05, ***P < 0.001.

We perturbed the expression of piR-017724 in SMMC-7721 and PLC/PRF/5 cells with mimics or inhibitors to analyze its biological function (**Figures 1D, E**). As shown by the CCK-8 assay, inhibition of piR-017724 expression resulted in a significant increase in the viability of SMMC-7721 and PLC/PRF/5 cells, while increasing piR-017724 expression decreased the viability of both cell lines (**Figures 2A, B**). Similarly, colony formation assays confirmed that piR-017724 reduced the number of SMMC-7721 and PLC/PRF/5 colonies (**Figures 2C, D**). This indicated that piR-017724 can inhibit the proliferation of HCC cells.

### The piR-017724 did not affect the apoptosis of HCC cells

**Figure 3:**
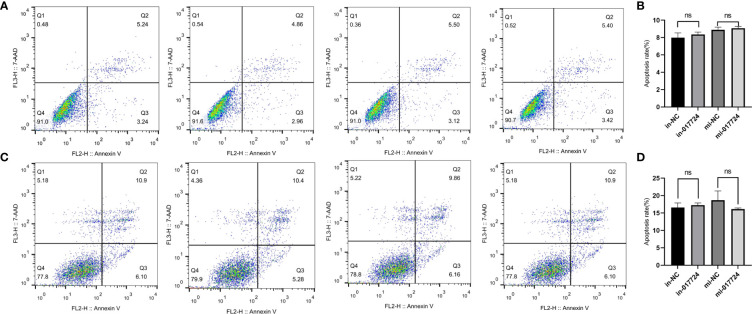
The piR-017724 did not affect the apoptosis of HCC cells. **(A, B)** Flow cytometry analysis was performed to detect the effect of hsa_piR_017724 knockdown and overexpression on cell apoptosis of SMMC-7721 cells. **(C, D)** Flow cytometry analysis was performed to detect the effect of hsa_piR_017724 knockdown and overexpression on cell apoptosis of PLC/PRF/5 cells. ns, Non Significance.

The apoptosis of SMMC-7721 and PLC/PRF/5 cells was detected by flow cytometry. The above results indicated that piR-017724 did not affect the apoptosis of HCC cells (**Figure 3**).

### The piR-017724 inhibits the migration/invasion ability of HCC cells

**Figure 4:**
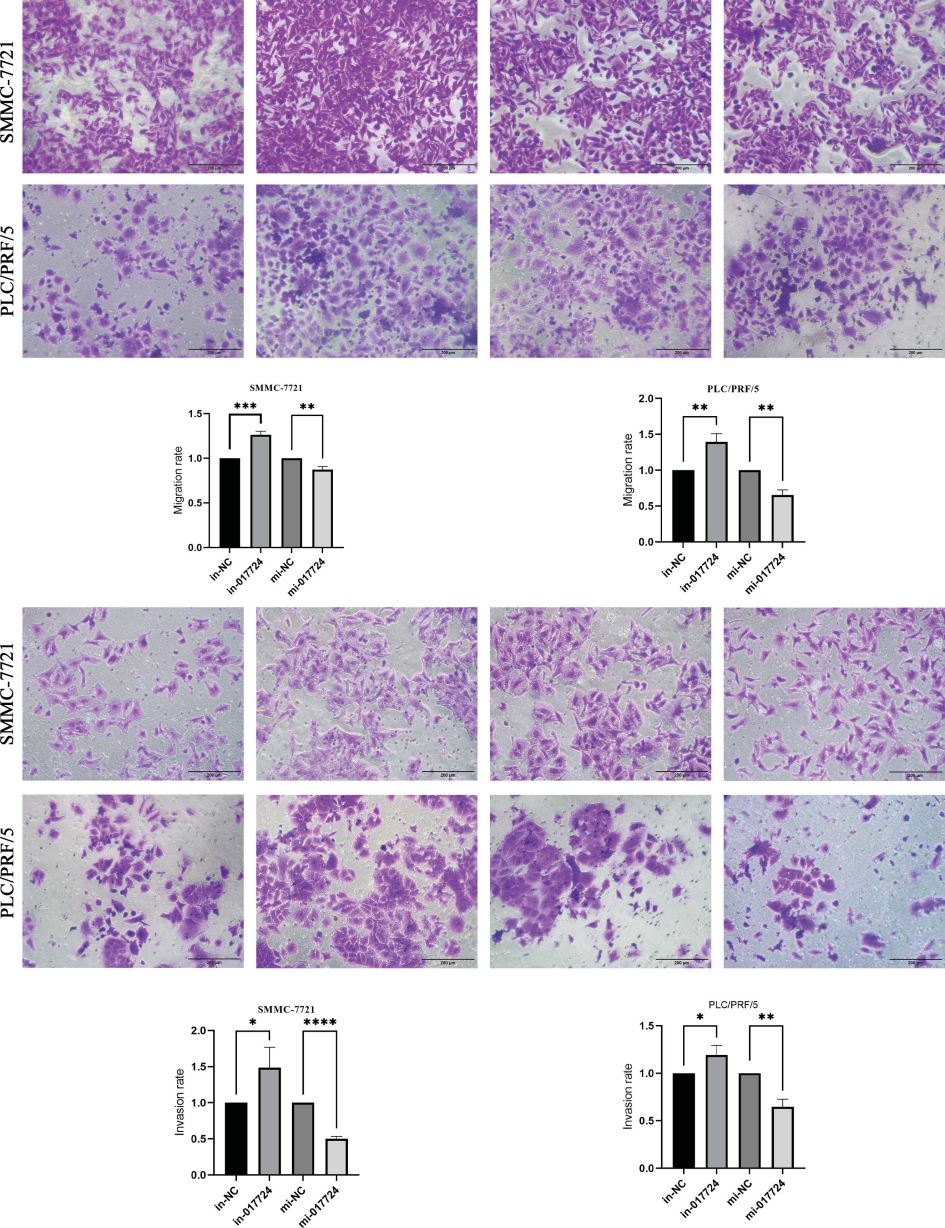
The piR-017724 inhibits the migration/invasion ability of HCC cells. **(A, B)** Transwell assays were used to examine the effect of hsa_piR_017724 knockdown and overexpression on cell migration of SMMC-7721 and PLC/PRF/5 cells. **(C, D)** Transwell assays were used to examine the effect of hsa_piR_017724 knockdown and overexpression on cell invasion of SMMC-7721 and PLC/PRF/5 cells. *P <0.05, **P <0.01, ***P <0.001, ****P <0.0001.

The ability to metastasize is assessed primarily through cell invasion and migration. The Transwell results showed that hsa_piR_017724 knockdown increased the migration ability of both cell lines compared with the in-NC group, while hsa_piR_017724 overexpression inhibited the migration ability of both cell lines compared with the mi-NC group (**Figures 4A, B**). We then tested the invasive ability of HCC cells by Matrigel transwell assay. Compared with the mi-NC group, hsa_piR_017724 overexpression inhibited the invasion of SMMC-7721 and PLC/PRF/5 cells, while cell invasion was enhanced in the hsa_piR_017724 inhibitor group compared with the in-NC group (**Figures 4C, D**). These results indicated that hsa_piR_017724 could inhibit the invasion and migration ability of hepatoma cells.

### The piR-017724 inhibits HCC development by silencing PLIN3

**Figure 5:**
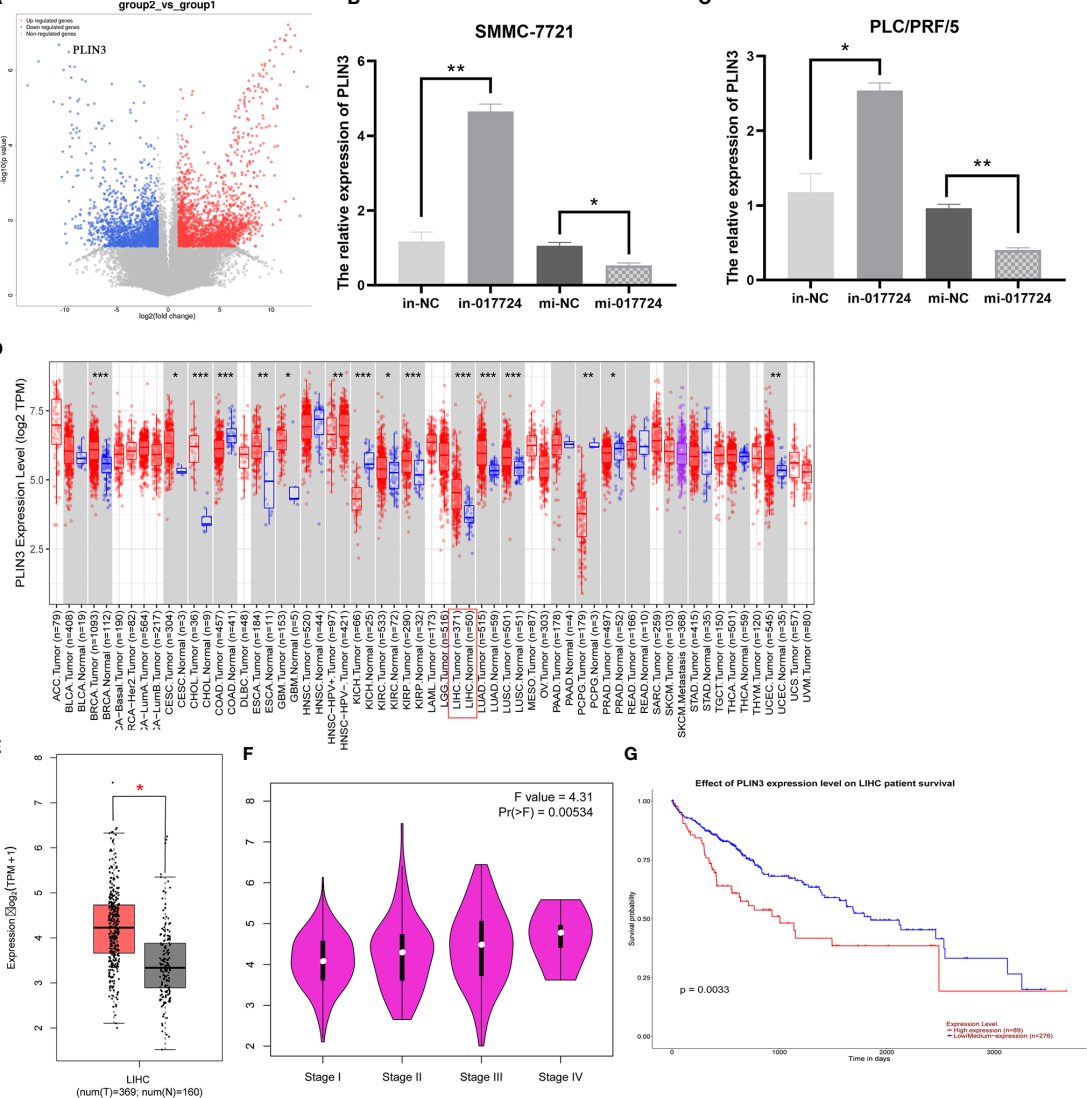
The piR-017724 inhibits HCC development by silencing PLIN3. **(A)** Prediction of piR-017724 targeted proteins by high-throughput sequencing technology. **(B)** qRT-PCR detected the expression of PLIN3 in SMMC-7721 cells after transfection. **(C)** qRT-PCR detects the expression of PLIN3 in PLC/PRF/5 cells after transfection. **(D)** Analysis of PLIN3 expression levels in HCC tissues on the TIMER2.0 database. **(E)** Analyze the expression level of PLIN3 in HCC tissues on the GEPIA database. **(F)** Analysis of the expression level of PLIN3 in different pathological stages on the GEPIA website. **(G)** The survival of patients in the PLIN3 high expression group was significantly lower than that in the PLIN3 low expression group. *P <0.05, **P <0.01, ***P <0.001.

Through high-throughput sequencing technology (**Figure 5A**) and bioinformatics analysis, the results suggest that the piR-017724 may potentially target GLRX3, HACD3, PLIN3, ZDHHC5, PRKAB2, GBF1 gene.

We then performed PCR verification: In SMMC-7721 and PLC/PRF/5 cells, compared with in-NC group, piR-017724 knockdown increased the expression of PLIN3, while compared with mi-NC group, piR-017724 overexpression decreased the expression of PLIN3 (**Figures 5B, C**). These results show that PLIN3 is a potential target of piR-017724 in HCC cells.

### PLIN3 expression is significantly upregulated in HCC and is associated with poor prognosis

We analyzed in the TCGA database and found that PLIN3 was highly expressed in HCC compared with adjacent normal tissues (**Figures 5D, E**). The correlation between the expression of PLIN3 and the pathological stage of cancer was observed through the “pathological stage map” module of HEPIA2. It was found that the expression of PLIN3 was related to the pathological stage. Cancer cases were divided into high expression group and low expression group according to the expression level of PLIN3, and the correlation between the expression of PLIN3 and the prognosis of HCC patients was studied using TCGA data set. It was found that the prognosis of HCC patients with high expression of PLIN3 was poor (**Figures 5F, G**).

Therefore, we speculate that piR-017724 inhibits the development of HCC by silencing PLIN3.

## Discussion

PIWI-interacting RNAs (piRNAs) are a new class of small ncRNAs (16, 17). To date, more than 20,000 piRNAs have been identified (18, 19). Some studies have shown that piRNAs are widely expressed in germ cells. Recently, high-throughput sequencing has shown that their signaling pathways are also active in somatic cells, particularly in human cancers (20–24), suggesting that they may play a role in carcinogenesis. piRNAs can affect multiple processes in cancer cells, including apoptosis, proliferation, migration, and invasion, by regulating gene expression at the transcriptional and posttranscriptional levels (21, 25). piRNAs have also been shown to exhibit pro-cancer or anticancer effects by directly binding to PIWI proteins (26). In recent years, by studying the roles of piRNAs and PIWI in cancer, scientists have found little evidence supporting a close link between piRNA/PIWI and various tumors. Aberrant expression of piRNAs is associated with various cancers and may play a pro-cancer or anticancer role in the occurrence, development and metastasis of cancer. The potential clinical implications of piRNA and PIWI as cancer diagnostic tools, therapeutic targets, and prognostic biomarkers are important. It has been reported that piRNAs are closely related to the malignancy of gastric cancer (27–29). The abnormal expression of piR-651 and piR-823 in normal and cancer tissues was closely related to the prognosis of gastric cancer and could be used as diagnostic tools or therapeutic targets for gastric cancer. Several studies have indicated that piRNAs play an important role in the pathogenesis of breast cancer and could be used as diagnostic markers (30–32). In renal cancer, studies have shown that piRNAs are closely related to the metastasis and prognosis of renal cancer (33–35). In colorectal cancer, piRNAs have important clinical significance as diagnostic tools and therapeutic targets (36–41). Compared with normal lung tissue, the expression of piR-55490 was decreased in lung cancer tissue, and inhibition of piR-55490 could promote the proliferation of lung cancer cells by inhibiting the activation of the mTOR pathway in lung cancer cells. This finding suggests that piR-55490 may have an anticancer effect in the development of lung cancer (24). piR-651 is involved in the occurrence, invasion and metastasis of non-small cell lung cancer and may be a potential cancer diagnostic tool (42–44).

Many studies have shown that Lipid droplets (LDs) are complex, dynamic and multifunctional organelles that play an important role in membrane transport, protein degradation, signal transduction and gene expression regulation (16). Members of the abdominal lipoprotein (PLIN) family are the most important LD-related proteins because they participate in the formation and degradation of LD. In addition, the PLIN family is composed of five member proteins, which have a conservative structure and the ability to bind intracellular LD (17, 18). Recent studies have shown that the abnormal expression of genes in the PLIN family may be a potential prognostic biomarker for various types of cancer, including sarcoma, hepatocellular carcinoma, renal carcinoma and breast cancer (19–22).

In our study, we first preliminarily confirmed the significant low expression of piR-017724 in HCC and its significant correlation with the malignant progression and poor prognosis of HCC patients, indicating that piR-017724 is a potential biomarker and therapeutic target for HCC; Subsequently, *in vitro* experiments confirmed that HCC cells can enhance their proliferation, invasion, and migration abilities by downregulating the expression of piR-017724. In addition, our study also found that piR-017724 may inhibit the development of HCC by targeting the silencing of PLIN3 expression. However, our current research can only determine the targeting effect of piR-017724 and PLIN3, so further research is needed in the future to explore the specific mechanism of piR-017724 involvement in the occurrence and development of HCC.

## Conclusion

In summary, This study identified piR-017724 as a new HCC biomarker that participates in the occurrence and development of HCC by targeting PLIN3 as a new mechanism, which provides new insights into the clinical application of piR-017724 in the treatment of HCC.

## Data availability statement

The original contributions presented in the study are included in the article/supplementary material. Further inquiries can be directed to the corresponding authors.

## Ethics statement

The studies involving human participants were reviewed and approved by the Ethics Committee of Affiliated Nantong Hospital 3 of Nantong University. The patients/participants provided their written informed consent to participate in this study.

## Author contributions

Study concept and design: ML, YN, W-HC and LC. Experiment and data acquisition: JW, H-XW, Y-JW, L-LJ and L-XY. Analysis and interpretation of data: PZ and L-LJ. Statistical analysis: PZ and JW. Drafting of the manuscript: Y-JW and JW. Funding: Y-LC and ML. All authors contributed to the article and approved the submitted version.
